# Investigation of Parameters Influencing Tubular-Shaped Chitosan-Hydroxyapatite Layer Electrodeposition

**DOI:** 10.3390/molecules26010104

**Published:** 2020-12-28

**Authors:** Mariusz Mąkiewicz, Radosław A. Wach, Katarzyna Nawrotek

**Affiliations:** 1 Department of Environmental Engineering, Faculty of Process and Environmental Engineering, Lodz University of Technology, Wolczanska 213 Street, 90-924 Lodz, Poland; mariusz.makiewicz@dokt.p.lodz.pl; 2 Institute of Applied Radiation Chemistry, Faculty of Chemistry, Lodz University of Technology, Wroblewskiego 15 Street, 93-590 Lodz, Poland; radoslaw.wach@p.lodz.pl

**Keywords:** chitosan, electrodeposition, kinetics, implant, tissue engineering

## Abstract

Tubular-shaped layer electrodeposition from chitosan-hydroxyapatite colloidal solutions has found application in the field of regeneration or replacement of cylindrical tissues and organs, especially peripheral nerve tissue regeneration. Nevertheless, the quantitative and qualitative characterisation of this phenomenon has not been described. In this work, the colloidal systems are subjected to the action of an electric current initiated at different voltages. Parameters of the electrodeposition process (i.e., total charge exchanged, gas volume, and deposit thickness) are monitored over time. Deposit structures are investigated by scanning electron microscopy (SEM) and Fourier-transform infrared spectroscopy (FTIR). The value of voltage influences structural characteristics but not thickness of deposit for the process lasting at least 20 min. The calculated number of exchanged electrons for studied conditions suggests that the mechanism of deposit formation is governed not only by water electrolysis but also interactions between formed hydroxide ions and calcium ions coordinated by chitosan chains.

## 1. Introduction

The electrodeposition process is one of the methods for manufacturing thin solid layers from an electrolyte solution using electric field. It has found application in various industries such as biology, biotechnology, biochemistry, chemistry, materials science and waste-water treatment [1,2,3]. Originally, the electrodeposition process was used for obtaining metal coatings. Recently, it has been employed in manufacturing hydrogel layers from synthetic or natural polymers. In the latter case, the systems reported in the literature are composed of collagen, silk, alginate or chitosan [4].

Both metal and hydrogel deposits have been applied in medicine for production of products that possess unique chemical and physical properties [5]. Depending on process parameters, electrodeposition products can be obtained in different forms such as electrode coatings, thin layers or cylindrical tubes [5,6]. In addition, the electrodeposition process is marked by mild conditions and low-cost apparatus. Although it is time consuming for some systems, it allows controlling the structure and morphology of deposited layers.

In 2016, our team reported a technique for obtaining tubular-shaped deposits from a chitosan-hydroxyapatite colloidal solution [7]. The novelty of this approach was the use of a stainless steel rod as an inner electrode. It allowed obtaining a tubular-shaped hydrogel structure, which was applied in peripheral nerve tissue engineering [8]. Peripheral nerves can regenerate spontaneously by themselves, but without proper guidance the healing process may be hindered, causing illnesses and dysfunctions (e.g., neuroma) [9,10]. The golden standard in reconstruction of disrupted peripheral nerve tissue is nerve grafts taken from autologous tissue (e.g., sural nerve, medial antebrachial cutaneous nerve and posterior interosseous nerves). Despite good clinical efficacy of autografting, the autologous tissue is limited and harvesting it causes donor site morbidity (i.e., neuroma formation, sensory loss, infection and surgical scarring). The promising alternative solution is inserting biocompatible tubular polymer implants [7]. The potential of our electrodeposited chitosan implants was studied in vitro and in vivo with satisfactory outcomes [8,11,12]. In addition, the electrodeposition process is highly reproducible [13]. Despite its high application potential, the influence of process parameters (i.e., voltage) on structural, chemical, and mechanical characteristics of chitosan deposits is inadequately studied [14,15].

It is known that cathodic electrodeposition of chitosan can undergo in the environment of pH less than 6.5, which facilitates forming a colloid. Electrochemical reaction of water reduction, which undergoes on the cathode, is presented as:

2H_2_O + 2e^−^ → H_2_ + 2OH^−^ (1)

Near the cathode, a sharp gradient of pH occurs. Chains of protonated chitosan are deposited on metal at pH 6.5 due to charge deprotonation of amino groups, which causes their precipitation according to the following equation [16,17]:

Chit-NH_3_^+^ + OH^−^ → Chit-NH_2_ + H_2_O (2)

There are some reports describing biological, physical, and chemical properties of chitosan-based deposits and their applications [6,18,19]. However, the literature is lacking in quantitative and qualitative explanation of mechanism of this process. A correlation between process parameters and their changes over time for electrodeposition from chitosan-hydroxyapatite colloidal solution in the cylindrical geometry has not been described. This knowledge would be helpful for manufacturing tubular implants with desired properties for customised nerves regeneration or other applications, where high control over chemical composition, mechanical parameters, and morphology is required.

The aim of this work is focused on the investigation of parameters influencing electrodeposition from chitosan-hydroxyapatite colloidal solution. The correlation between the total charge exchanged, hydrogen liberation, and deposit thickness over time was described. In addition, apparent density and water content was calculated for the investigated range of voltages. In order to qualitatively characterise the structure of fabricated deposits, scanning electron microscopy and Fourier-transform infrared spectroscopy were employed. The obtained results will be a handful tool for manufacturing an implant with desired properties for an individual medical case, for instance a person suffering from peripheral nerve tissue disruption.

## 2. Results

In order to determine the parameters of particles in colloidal solutions, measurements of particle size and zeta potential were performed. The results are collected in Table 1. Chitosan in acidic conditions can be degraded; however, this process is slow, and significant changes can be observed not earlier than after 10 days of the solution storage [20,21].

**Figure 1:**
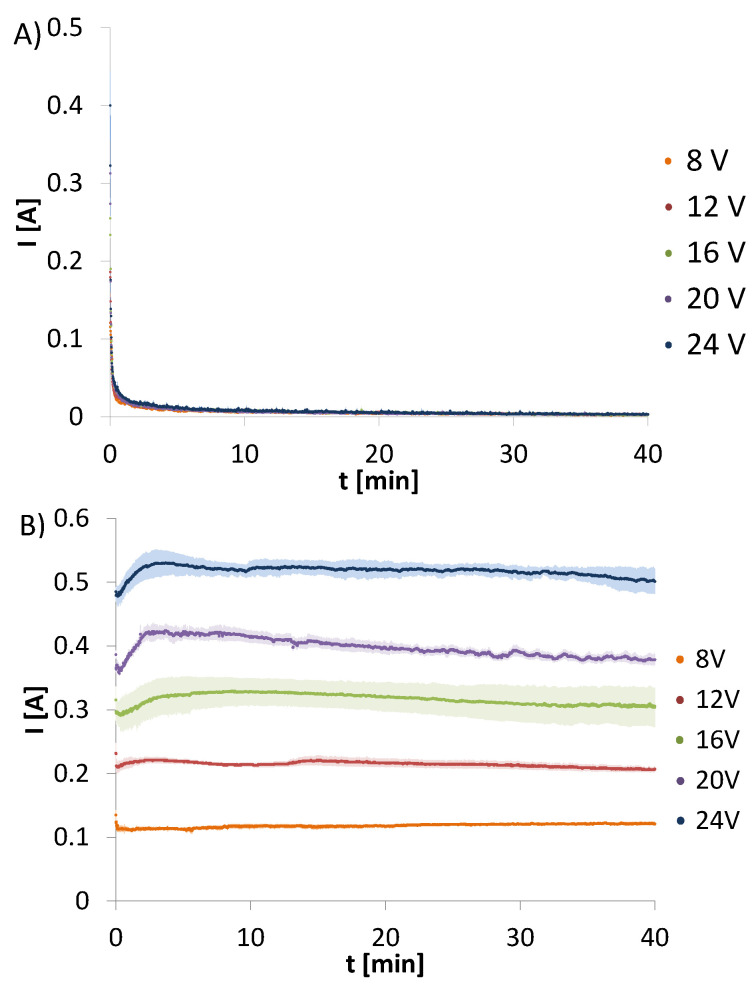
Amperage changes over time for electrodeposition from (**A**) chitosan and (**B**) hydroxyapatite colloidal solutions initiated at different voltages. Data are shown as a mean ± standard deviation (n = 3).

Amperage changes over time were recorded for electrodeposition from chitosan, hydroxyapatite and chitosan-hydroxyapatite colloidal solution. The changes of amperage for CHcs recorded for the initial voltages set at 8, 12, 16, 20, or 24 V are presented in Figure 1A Electrodeposition form CHcs does not result in a solid deposit. Only a very thin semi-stiff hydrogel layer was observed. The changes of amperage over time for all initial voltages are the same and show rapid decrease.

Figure 1B shows the changes of amperage for HApcs recorded for the initial voltages set at 8, 12, 16, 20, or 24 V. The process does not lead to any deposit on the electrode and the plots are characterised by a constant current.

**Figure 2:**
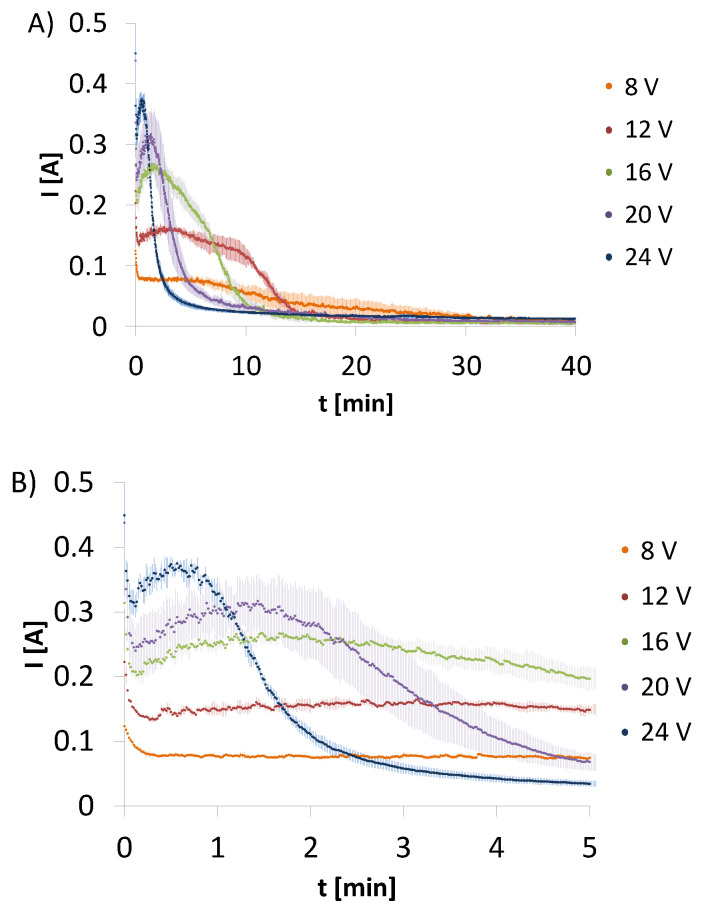
Amperage changes over (**A**) 40 min and (**B**) 5 min for electrodeposition from chitosan-hydroxyapatite colloidal solution initiated at different voltages. Data are shown as a mean ± standard deviation (n = 3).

In contrast to the above electrodeposition processes from CHcs and HApcs, changes of amperage over time for electrodeposition form CH-HApcs were observed. The amperage was recorded for the initial voltages set at 8, 12, 16, 20, or 24 V (Figure 2). All plots have similar shapes, i.e., a rapid amperage drop is observed during the first 10 s of the process. The higher initial voltage, the higher maximum value of amperage is recorded. After the initial period of 10 s, an increment in amperage can be observed to continue until the maximum value is reached. However, at the initial voltage, equal to 8 V, the local maximum is not observed. In addition, the lower the voltage, the longer the time required for reaching the maximum value.

In order to obtain the kinetics of the total electric charge exchanged, volume of hydrogen evolved, and increase in deposit thickness, the following respective measurements and calculations were made.

In the first step, for the obtained data, the electric charge ($Q_{t})$ exchanged during electrodeposition process was calculated. For this purpose, the definite integral of I(t) over [0, t] was employed:

(3) $$Q_{t}=\int_{0}^{t}I\left(t\right)dt$$

where *I*—amperage (A) and *t*—time (s).

**Figure 3:**
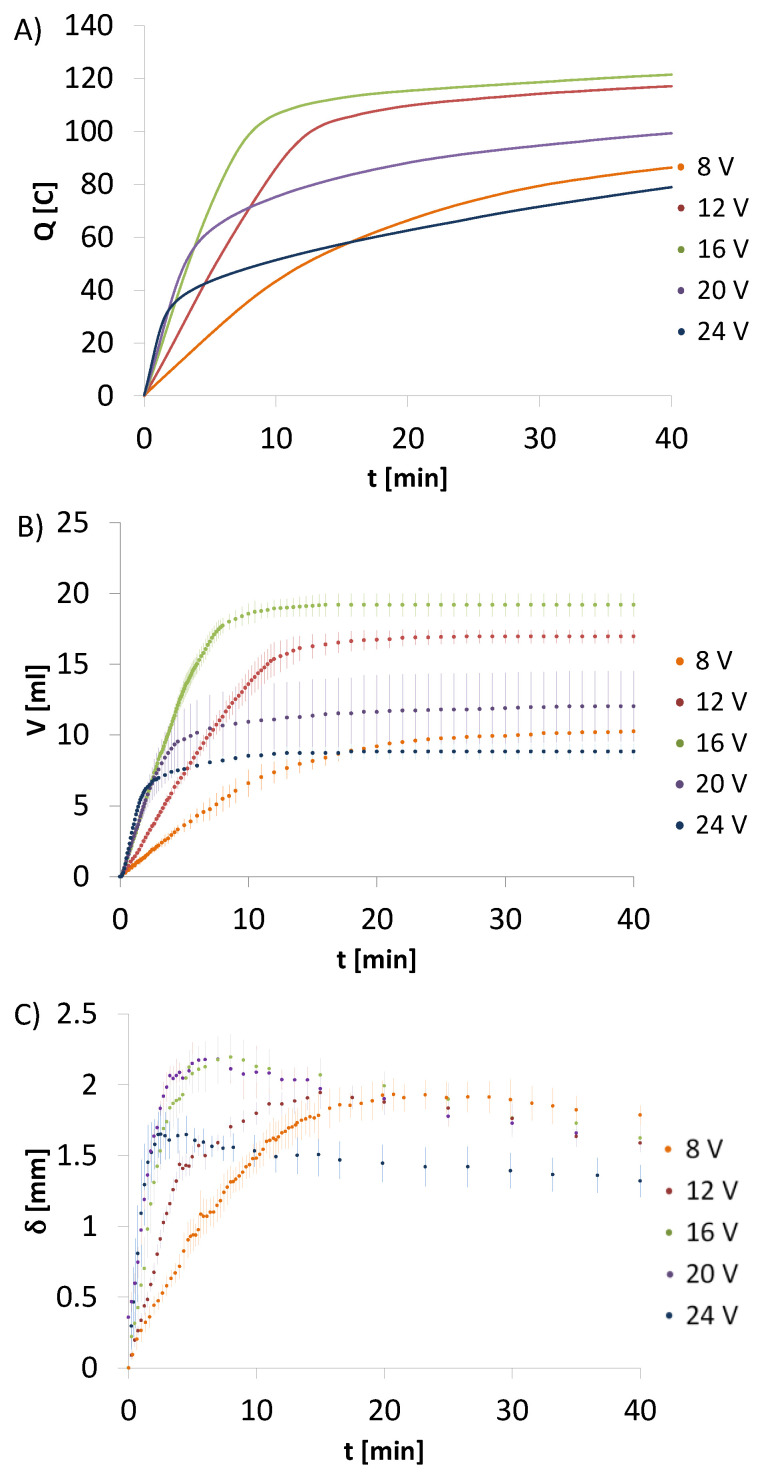
Change of (**A**) charge, (**B**) hydrogen volume, and (**C**) deposit thickness over time for electrodeposition from chitosan-hydroxyapatite colloidal solution initiated at different voltages. Data are shown as a mean ± standard deviation (n = 3).

The total charge exchanged during 40 min of the electrodeposition process was calculated too (Table 2). The calculated values for CHcs are lower than for CH-HApcs. The total electric charge takes the highest values for HApcs. Analysing the results for CH-HApcs, it can be noticed that the electric charge rises rapidly to the point where amperage reaches the minimum value (Figure 3A). Moreover, the process for the initial voltage set at 16 V generates the highest total charge, whereas the processes initiated at voltages of 8 and 24 V induce similar values of the total charge.

In the next step, the total amount of gas produced during electrodeposition process was determined. For this purpose, the ideal gas equation was employed. The calculations were conducted using the values of hydrogen volume collected in the eudiometer and are presented in Table 2. The calculated values of evolved hydrogen for CHcs are lower than for CH-HApcs. For HApcs, the hydrogen volume takes the highest values. In this system, the gas volume decreases for the initial voltages set at 16, 20, and 24 V. In addition, the constant hydrogen volumetric flow is observed. Since there is only a slight amperage decrease after 40 min of the process, the total electric charge received is higher than for the system containing only chitosan.

In the final step, the deposit mass, dry deposit mass, water content, and apparent density for the particular electrodeposition processes was measured (Table 2). Figure 3C presents the rate of deposit thickness increase versus time.

**Figure 4:**
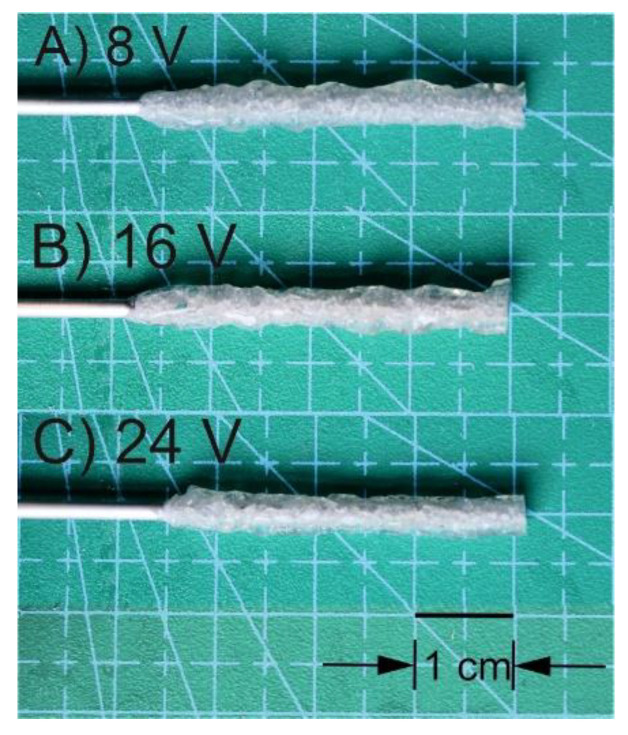
The photographs of deposits obtained from chitosan-hydroxyapatite colloidal solutions for the electrodeposition process initiated at voltages: (**A**) 8, (**B**) 16, and (**C**) 24 V.

The photographs of deposits obtained from chitosan-hydroxyapatite colloidal solution for the electrodeposition process initiated at voltages 8, 16, and 24 V are shown in Figure 4. The applied reactor and initial volume of colloidal solution enabled receiving structures with a length of 38 ± 2 mm. They could be easily removed from the rod. It can be noticed that there is no significant influence of the initial voltage on the average deposit thickness for the process lasting at least 20 min, with the exception for 24 V, where the conduit is less than 0.5 mm thinner than for other cases.

**Figure 5:**
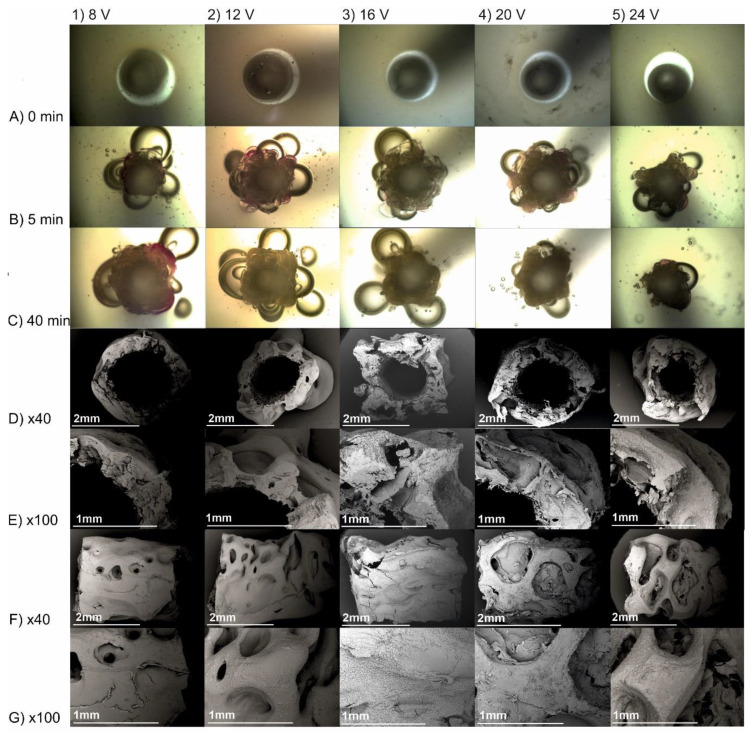
Optical microscope images of deposits obtained from chitosan-hydroxyapatite colloidal solution for the electrodeposition process initiated at (**1**) 8, (**2**) 12, (**3**) 16, (**4**) 20, and (**5**) 24 V after (**A**) 0, (**B**) 5, and (**C**) 40 min. SEM images of (**D**) transverse cross-section, (**E**) 100× magnification of the lumen surface, (**F**) longitudinal view, and (**G**) 100× magnification of the outer surface obtained after 40 min.

In order to perform detailed studies of gain in deposit thickness over time, the electrodeposition process was recorded under the optical microscope (Figure 5A–C). In every case, hydrogen was evolved, and its amount was quantified. The violet colour of chitosan-hydroxyapatite layer was the consequence of interaction of OH^−^ ions with phenolphthalein. The resulting alkaline environment facilitated chitosan deposition. After 30 min of the process, only single bubbles were observed. The higher the voltage, the higher the amount of hydrogen that is evolved (Table 2). The mass of deposits just after their preparation and the dry ones for electrodeposition run for 40 min at 8, 12, 16, 20, and 24 V was equal to 0.380 ± 0.063 and 0.074 ± 0.011, 0.416 ± 0.013 and 0.088 ± 0.007, 0.331 ± 0.021 and 0.082 ± 0.004, 0.319 ± 0.023 and 0.087 ± 0.005, and 0.307 ± 0.022 and 0.081 ± 0.003 g, respectively. The lower the induced voltage, the higher the water content. Moreover, the apparent density is higher for higher voltages (Table 2). In order to qualitatively assess structural changes caused by liberating hydrogen within the bulk of structures, scanning electron microscopy was employed (Figure 5D–G). The higher the voltage, the higher the irregularity of the implant outer surface.

**Figure 6:**
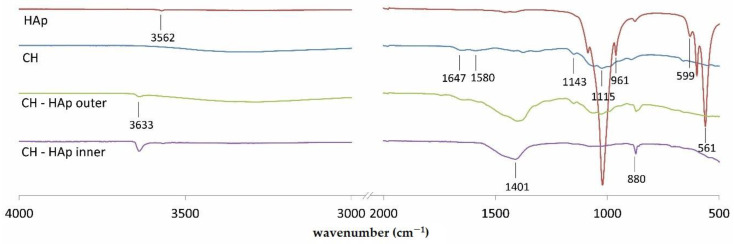
FTIR spectra of native chitosan, hydroxyapatite, and surfaces (inner and outer) of deposit obtained from the chitosan-hydroxyapatite colloidal solution for the electrodeposition process initiated at 12 V after 40 min.

In Figure 6, FT–IR spectra of hydroxyapatite, chitosan, and surfaces (inner and outer) of CH-HAp deposit are presented in order to understand interactions between chitosan and hydroxyapatite created in implants. The peaks characteristic for chitosan are present at: 1647 (–C=O stretching mode), 1580 (–NH_2_ bending mode), and three peaks located in the range from 1020 to 1140 cm^−1^ (C-O-C stretching asymmetric and symmetric mode). The spectrum of hydroxyapatite shows characteristic peaks associated with ${\mathrm{PO}}_{4}^{3-}$ at 1020, 961, and 561 cm^−1^. Moreover, the signal observed at 3562 cm^−1^ denotes the stretching mode of -OH bond [7,22,23,24]. The strongest characteristic ${\mathrm{CO}}_{3}^{2-}$ bands are visible at 1420 cm^−1^. Carbonate is often present in hydroxyapatite as a production residue [22]. The spectra of inner and outer surfaces of implants show well-pronounced peaks at 1401 and 1387 cm^−1^, respectively. They demonstrate peaks characteristic for chitosan at 1647, 1580, 1143, and 1015 cm^−1^. The weak peak assigned to lactic acid at 1749 cm^−1^ (attributed to carbonyl of ester or carboxylic groups) is detected in the spectrum of outer surface. Moreover, CH-HAp inner and outer surfaces show peaks at 3633 and 880 cm^−1^.

## 3. Discussion

In recent years, researches have investigated a variety of products which can be applied in nerve tissue regeneration [25]. Nevertheless, only a few papers bring up the topic of tubular-shaped layer electrodeposition [26,27]. In addition, up to now, the quantitative and qualitative characterisation of electrodeposition process from polymeric colloidal solution has been poorly described. Taking into account the high application potential of electrodeposited structures in the production of personalised implants, particularly for peripheral nerve tissue engineering, the presented work was focused on investigation of parameters influencing the tubular-shaped layer electrodeposition from chitosan-hydroxyapatite colloidal solution.

The work protocol encompassed three steps. Firstly, the properties of the prepared colloidal systems (i.e., particle size and zeta potential) were determined. Secondly, the parameters of the electrodeposition process were characterised. In this step, the change of amperage and deposit thickness over time were studied. In addition, the amount of evolved hydrogen was monitored. The final step of the work was focused on structural characterisation of deposits. The water content and apparent density was determined for CH-HAp implants. The structures were also qualitatively assessed based on the results of morphology by SEM and chamical composition by FTIR.

The obtained results of z-average particle size confirm colloidal character of the studied solutions, since the particle sizes of respective molecules are lower than 1 nm. The average size of particles in CH-HApcs is bigger than in CHcs and HApcs. This observation suggests formation of new complexes with higher positive zeta potential for the complex system. The new interactions might be the result from chelation of hydroxyapatite-derived species by chitosan chains [28]. Moreover, it is known that in the range of pH values between the pK_a_ of lactic acid (3.8) and the one of chitosan (~6), an ionic pair between RCOO^−^ and protonated chitosan (−NH_3_^+^) is formed [29]. It was also shown that the lactic acid counter-anion interacts strongly with the chitosan chains [30]. The resulting high zeta potential facilitates movement of particles towards the cathode.

Analysing the results for change of amperage over time, a sharp drop within first 10 s of electrodeposition process is observed. This might result from an adhesion of hydrogen bubbles to the cathode surface. This gas layer forms a resistancebarrier, which limits the current flow between the electrodes. Positively charged particles dispersed in the system move in the electric field towards the cathode and negative hydroxide ions, which occurs as a product of water electrochemical reduction, are repulsed. As the zeta potential is the highest for CH-HApcs particles, their movement towards the cathode is strongly favoured. Simultaneously, calcium ions derived from hydroxyapatite might be involved in the formation of calcium hydroxide (Ca(OH)_2_) with hydroxide ions created in electrochemical reaction of water reduction. This phenomenon takes place at the deposit-colloid interface. Simultaneously, –NH_3_^+^ groups of chitosan are also deprotonated by hydroxide ions, resulting in neutral amino groups. Then, calcium hydroxide might be immediately chelated by –NH_2_ groups, forming coordination bonds with free electron pairs on nitrogen atoms. As a result, this phenomenon initiates deposition of the created complex on the cathode [28]. The forming layer starts gradually replacing hydrogen bubbles and continuously serves as a base for incoming chitosan chains. Moreover, the initial amperage increase observed for HApcs (Figure 1B) suggests the promoting effect of this compound on the formation of hydroxide ions. Their formation can be observed with the colour change of phenolphthalein to purple near the cathode. The concentration of hydroxide ions for CH-HApcs is bigger than for CHcs, which causes growth of amperage until a local maximum is reached. As the deposit thickness increases, the resistance of the solution increases too. Upon reaching the maximum values, a drop in amperage is observed. Transport of hydroxide ions originated form water electrolysis and calcium ions derived from hydroxyapatite dissociation is hindered by a deposited layer. Therefore, the formation of calcium hydroxide, which can promote further thickness growth, is inhibited. For lower voltages, the maximum value of amperage is reached later because of slower increase of implant thickness. The lower the set voltages, the lower the value of the maximum amperage. However, for the process initiated at 8 V, there is no maximum observed and only a plateau value exists, which slightly decreases after 20 min. The observed fluctuations of amperage are caused by hydrogen formation, which changes the resistance of the system over time. The high viscosity of CH-HApcs allows for the adhesion of bubbles to implant until they reach the critical size for detachment. The recorded changes of amperage over time as well as a lack of solid deposits for CHcs and HApcs suggest that chitosan-hydroxyapatite electrodeposition results from the interaction between chitosan and hydroxyapatite-derived species.

The amperage plot for the CH electrolyte presents a sharp drop of values over time and solid product is not obtained. Moreover, the amount of hydrogen formed during the process is lower than the one collected for CH-HAp electrolyte. Nevertheless, a denser layer of suspension observed near the cathode after process initiation can cause a resistance increase. Deposition of pure chitosan layer is possible in HCl solution. In the literature, the moving front model was proposed for electrophoretic deposition of chitosan dissolved in this acid. It is based on three assumptions. Firstly, the degree of deprotonation is equal to the total electric charge transferred to the electrode. Secondly, chains in the gel area are totally deprotonated, while those in the solution are totally protonated. Finally, the concentration of chitosan is constant in the entire system. In this model, if the pH gradient front and gelation front are co-localised, the ratio of deprotonation and chitosan deposit formation are equal [31]. It was confirmed that under constant current density, in pair side-wall electrode system, the thickness gain is linear and increases with the increase of the set current density [17]. However, it slightly decreases upon chitosan depletion after 100 s. The observation of RHS-rodamine-tagged chitosan electrodeposition indicated that in the area of the gradient, a depletion of chitosan concentration is observed. This is the effect of polymer shrinkage after neutralisation of positive charge on its amino groups. It was postulated that movement of protonated chitosan molecules is controlled by electric field and a concentration gradient [17]. It is known that the rate of electrodeposition depends also on pH of solution used for process. The lower the pH value, the higher the deposition rate [32].

The electrodeposition of hydroxyapatite in lactic acid water solution undergoes at almost constant amperage over time, with slight increase for voltages higher than 8 V at the beginning of the process. In this process, the flow rate of hydrogen is almost steady; however, deposition of a solid layer is not observed. This suggests that hydroxyapatite facilitates the current flow during the initial period of the co-deposition process, while migrating chitosan particles and hydrogen bubbles adhered to the cathode. The positive zeta potential of hydroxyapatite in the wide range of pH (3–10) facilitates its movement to the cathode. This might result from the presence of calcium-based ions (e.g., Ca^2+^, CaOH^+^, CaH_2_PO_4_^+^). However, the deposition of hydroxyapatite from aqueous solution is not possible because of the adsorption of water molecules to it [33]. It was shown that hydroxyapatite can be deposited at the cathode only in non-water solvents, e.g., isopropanol [14,34]. Calcium-based positive ions migrating towards cathode are connecting with negative charged ions OH^−^ and PO_4_^3−^ to form hydroxyapatite precipitate (Ca_10_(PO_4_)_6_(OH)_2_) [35,36].

For the chitosan-hydroxyapatite colloidal solution, the number of electrons exchanged (z) can be determined form the following equation:

(4) $$z=\frac{Q_{t}}{n_{H_{2}}F}\;$$

where *Q_t_*—total electric charge exchanged during electrodeposition over 40 min (C), $n_{H_{2}}$—amount of evolved hydrogen over 40 min (mol), and *F*—Faraday constant (C/mol).

The obtained values are presented in Table 3. The integer number calculated for all voltages can be approximated to 2. These calculations confirm that during electrochemical reaction taking place on electrode two electrons are exchange, which does not deny the correctness of the water electrolysis described earlier (Equation (1)). At the outer electrode, which serves as an anode, gas evolution and corrosion of iron was not observed. Nevertheless, oxidation of iron to the ions Fe^2+^ might take place. On the other hand, the calculated number of electrons exchanged also suggests the presence of other reactions.

Pictures taken with an optical microscope (Figure 5) during the process show a sharp pH drop at the interface of the deposit and solution as indicated by a violet colour of phenolphthalein only at the closest area of the cathode. This observation confirms that electrodeposition results from deprotonation of positive-charged chemical particles. An analysis of the average deposit thickness over time indicates its constant increase during the initial period. Upon reaching the maximum value of thickness, shrinkage of deposit is observed. This phenomenon might be caused by hydrogen detaching from the bulk of the deposited layer. In addition, it might result from a lack of new hydroxide ions. The secondary protonation of chitosan chain is hindered. As a consequence, it leads to the partial dissolution of the deposited layer exposed to the direct contact with the electrodeposition solution. Comparing changes in thickness, the total electric charge exchanged, and hydrogen volume, it can be observed that after 20 min, the deposit does not increase in thickness, a smaller amount of charge is exchanged, and hydrogen evolution is nearly entirely suppressed. Moreover, almost steady value of current is reached. It suggests that the charge transfer induces electrochemical reduction of water, which provides hydroxide ions, which in turn cause the hydrogel deposition.

The lower the set voltages, the lower irregularity of the external surface of the deposit. It is an effect of the slower rate of hydrogen formation and less amperage fluctuation during the process. Optical microscope photographs taken during electrodeposition (Figure 5) show a higher irregularity of deposited material on the cathode rod at the beginning of the process. Taking into consideration the average mass of the obtained conduits from CH-HApcs, an insignificant influence of voltage on the final dry product weight can be found. Moreover, looking at the thickness gain rate, the highest local maximum can be observed for 16 and 20 V. Analysing the total electric charge exchanged during the process, the received values are the highest for 12 and 16 V. The water content is the highest for 8 V, whereas the apparent density is the lowest. The mass of the deposit (m_h_ and m_d_) is the highest for 12 V. Moreover, the total amount of evolved hydrogen is the highest for 16 V. In addition, the inhibiting effect of a higher voltage on hydrogen liberation can be noticed. For 24 V, the volume of evolved gas is two times lower than for 12 and 16 V.

Comparison of FTIR spectra of inner and outer surfaces of CH-HAp deposit with the ones of chitosan and hydroxyapatite indicates creation of heterogeneous chemical structure. Both the outer and inner surface spectra show signals at 3633 cm^−1^ that are characteristic for the stretching mode of the -OH bond. They are also characterised by peaks located in the range from 1020 to 1140 cm^−1^, which can be assigned to chitosan. However, for the outer surface, these signals are more pronounced. Weak signals, characteristic for phosphate ions as well as lactate, indicate that these moieties created interactions with chitosan chains. Considering the possible interactions between the ingredients of the deposit, it can be concluded that Ca^2+^ ions originating from hydroxyapatite might interact with dissociated carboxyl groups of lactic acid or amino groups of chitosan [7]. The intensity of the peaks at 3633, 1401 and 880 cm^−1^ is higher for the inner surface of the CH-HAp deposit than for the outer one. It may suggest that the ratio of hydroxyapatite-derived species to chitosan chains is higher for the inner surface.

Co-deposition of chitosan and hydroxyapatite is possible while hydroxyapatite-derived species are chelated by previously deprotonated amino groups, which can be confirmed by higher values of zeta potential for particles in CH-HApcs. After water electrolysis, during which hydroxide ions are produced, chitosan chains bonded with calcium ions connect with them and form porous semi-solid gel on the cathode. Hydroxyl groups can be observed also on outer sidewall of deposit, although in smaller amounts (comparing FTIR signals for inner and outer surface). This can also be an explanation of further inhibition of chitosan deposition. While hydroxyl groups are connected to the gel, they cannot bind to other chitosan chains. Moreover, subsequent production of hydroxide ions on electrode is hindered because of a prior deposited layer. The formed layer can also obstruct negative charge of the electrode for positive charged particles of chitosan and hydroxyapatite, which as a consequence leads to the inhibition of particle migration towards the cathode.

SEM images of the obtained deposits show higher surface irregularities for samples obtained at higher voltages. The water content for deposits obtained from electrodeposition processes is higher for deposits prepared at lower voltages, whereas the apparent density is higher for deposits prepared at higher voltages. The appropriate combination of the parameters influencing deposit porosity is a crucial factor in electrodeposition, which ensures the transport of hydrogen and ions between the solution and cathode.

## 4. Materials and Methods

### 4.1. Materials

Chitosan (CH, type: 85/500) was obtained from Heppe Medical Chitosan GmbH (Halle, Germany). Degree of deacetylation (DD), viscosity (µ, 1% in 1% acetic acid, 20 °C), and viscosity average molecular weight (Mv) are equal to 82.6–87.5%, 351–750 mPas, and 472 kDa, respectively. Lactic acid (LA) and hydroxyapatite (HAp, nanopowder, <200 nm particle size) were purchased from Merck KGaA (Darmstadt, Germany).

### 4.2. Methods

#### 4.2.1. Chitosan, Hydroxyapatite, and Chitosan-Hydroxyapatite Colloidal Solutions Preparation

In order to study the parameters influencing electrodeposition process, the following systems were prepared: chitosan colloidal solution (CHcs, pH 2.78), hydroxyapatite colloidal solution (HApcs, pH 2.50), and chitosan-hydroxyapatite colloidal solution (CH-HApcs, pH 2.93). Briefly, 0.8 g of CH, 0.1 g of HAp, or 0.8 g of CH and 0.1 g of HAp were dispersed in 100 mL of 3% *w*/*w* LA, respectively. The CH-HAp solution composition was chosen based on its potential for production of deposits with the thickness at least 1.5 mm, which facilitates the measurements described in subsequent paragraphs. The dispersions were stirred (under moderate rotations) until complete dissolution for 24 h at room temperature.

#### 4.2.2. Particle Size and Zeta Potential Measurements

In order to characterise the inherent properties of constituents of the solution for deposition, the size of the particles and the zeta potential were measured, respectively, by dynamic laser scattering and by electrophoretic light scattering methods using Zetasizer Nano ZS (Malvern Instruments, Malvern, UK). The following parameters were used: laser wavelength—633 nm (He-Ne laser line) and scattering angle—173°. Measurements were performed at 25.0 ± 0.1 °C for the following solutions: CHcs, HApcs, and CH-HApcs. All solutions were diluted (1:4) and filtered through 1.2 micron pore size filters (Sartorius) for 2 h and 30 min before the measurements. The same acid solution for preparation of each dispersion was used as a diluent [30]. A mean value of at least three independent measurements was determined and a standard deviation was calculated.

#### 4.2.3. Amperage and Volume of Hydrogen Evolved during Measurements

**Figure 7:**
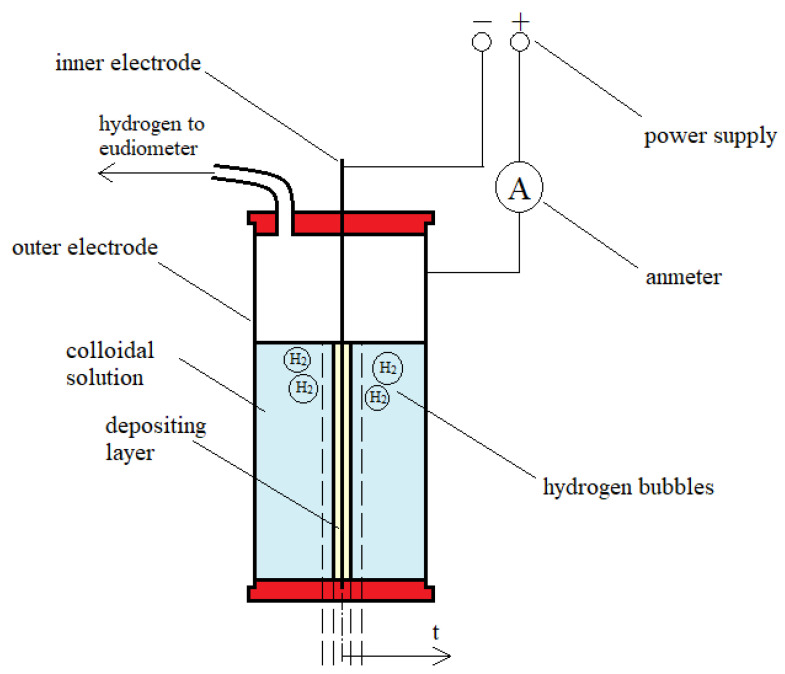
Schematic picture of reactor for tubular-shaped layer electrodeposition.

In order to examine the parameters of electrodeposition, 22 mL of CHcs, HApcs, or CH-HApcs was poured into a stainless steel tank (employed as an anode) of an in-house designed reactor (Figure 7). The inner stainless steel cathode applied had a diameter of 2 mm and the outer one had an inside diameter of 30 mm. The reactor was tightly closed with a cap equipped with an outlet connected to a eudiometer for collecting evolved hydrogen. The reactor was connected with a power supply and an ammeter. Data of amperage were collected by PC-Link software (Link Engine Management Ltd., Christchurch, New Zealand). A mean value of at least three independent measurements was determined and a standard deviation was calculated. The electrodeposition process was conducted for 40 min at constant temperature of 25 °C ± 0.1 °C with the initial voltage set at 8, 12, 16, 20, or 24 V. The applied time of the process was sufficient for reaching maximum deposition yield. The kinetics of hydrogen gassing was tracked using a digital camera. A mean value of at least three independent measurements was determined and a standard deviation was calculated. In addition, the volume of hydrogen was registered using the eudiometer and calculated by applying the ideal gas equation.

#### 4.2.4. Gain in Deposit Thickness Measurements

In order to track the deposit-solution interface over time, two drops of phenolphthalein (1% solution in ethanol, POL-AURA, Różnowo, Poland) was added to 10 g of CH-HApcs. The solution was mixed (under moderate rotations) for 10 min. Then, it was poured into the reactor tank without the cap. The reactor was installed under the optical microscope (Bresser GmbH, Rhede, Germany) equipped with an advanced ICD 10–16× microscope camera. After initiating of the electrodeposition, the recording of gain in deposit thickness was continued for 40 min. A mean value of three different measurements was determined and a standard deviation was calculated for all measurements. The mean thickness of deposit (δ) was calculated form the following equation:

(5) $$\delta =\frac{\sqrt{\frac{4S}{\pi }}-d_{e}}{2}$$

where *S*—the area of the cross-section of the deposit measured using ImgeJ software on the basis of a picture taken during the electrodeposition process [mm^2^] and *d_e_*—diameter of the cathode (*d_e_* = 2 mm).

#### 4.2.5. Structural Studies

In order to determine the water content of hydrogels, the deposits were divided into two groups: hydrated and dry. The mass of hydrated specimens was measured just after their preparation. To determine the mass of dry ones, deposits were placed at 25 °C ± 0.1 °C for one week. A mean value of three different measurements for both measurements was determined and a standard deviation was calculated. The water content was determined using the following equation:

(6) $$X=\frac{m_{h}-m_{d}}{m_{h}}\cdot 100\%$$

where *X*—water content (%), *m_h_*—mass of deposit (g), and *m_d_*—mass of dry deposit (g). A mean value of three different measurements was determined and a standard deviation was calculated.

The apparent density was calculated from the following equation [37]:

(7) $$\rho_{app}=\frac{4m_{d}}{\pi L\left[{\left(d_{e}+2\delta \right)}^{2}-d_{e}^{2}\right]}$$

where $\rho_{app}$—apparent density (g/cm^3^), *m_d_*—mass of dry deposit [g], *δ*—mean thickness of deposit (mm), *d_e_*—diameter of the cathode (*d_e_* = 2 mm), and *L*—length of conduit (*L* = 38 mm).

Samples for the SEM and FTIR analyses were taken immediately after fabrication. Firstly, deposits were dehydrated by placing in an exiccator containing water and ethyl alcohol (1:1) for 24 h. Then, the samples were removed from the exiccator and left open for 24 h to evaporate ethanol. Such a drying procedure allows keeping the shape of hydrogel structures. Dry conduits were cut into specimens with a length of 5 ± 0.5 mm for further examination. Scanning electron microscopy photographs of gold-coated deposits were taken with a Hitachi TM-1000 microscope (Hitachi, Ltd., Tokyo, Japan). SEM images were taken for the transverse cross-section, 100× magnification of the lumen surface, longitudinal view, and 100× magnification of the outer surface. FTIR spectra were obtained using Nicolet™ iS50 FTIR Spectrometer (Thermo Scientific, Madison, WI, USA) equipped with a diamond ATR. The spectra were collected over the range of 500–4000 cm^−1^ for the inner and outer surfaces of the deposits.

## 5. Conclusions

The application of an electric current to a chitosan-hydroxyapatite acidic colloidal solution results in the deposition of the structure composed of chitosan chains and hydroxyapatite-derived species on the cathode. Its morphology is dependent on the initial voltage set. The higher the set voltage, the lower the water content. The opposite trend is observed for the apparent density and surface irregularities of the deposits. For processes lasting for at least 20 min, the resulting deposit thickness is independent on the voltage in the employed reactor geometry and dimension. In future studies, we plan to determine the influence of the cathode diameter, distance between electrodes, concentration of solution components, and temperature, in order to identify the most important ones. The calculated number of exchanged electrons for the studied voltages for electrodeposition from chitosan-hydroxyapatite colloidal solution in the cylindrical geometry suggests that the deposit formation is governed not only by water electrolysis but also interactions between formed hydroxide ions and calcium ions coordinated by chitosan chains by chelation mechanism. The obtained relationship between the voltage and resulting deposit characteristics (chemical as well as mechanical) is a handful tool for obtaining tubular implants with desired properties for an individual medical case suffering from disruption of cylindrical tissues or organs, especially peripheral nerve tissue.

## Figures and Tables

**Table 1:** Particle size and zeta potential of chitosan, hydroxyapatite, and chitosan-hydroxyapatite colloidal solution.

Colloidal Solution	Z-Average Size (nm)	Polydispersity Index	Z-Potential (mV)
CHcs	942 ± 63	1	61.2 ± 5.3
HApcs	19 ± 4.7	0.92	21 ± 10
CH-HApcs	1281 ± 335	0.81	65.2 ± 6

**Table 2:** Quantitative characteristics of electrodeposition from chitosan, hydroxyapatite, and chitosan-hydroxyapatite colloidal solutions. Data are shown as mean values (n = 3). Q_t_—total electric charge, $n_{H_{2}}$ —amount of produced hydrogen, X—water content, ρ **_a_**—apparent density.

U (V)	8	12	16	20	24
**CHcs**					
Q_t_ (C)	14.3	15.6	16.8	16.0	18.6
$n_{H_{2}}$ (μmol)	73.7	49.8	66.4	70.4	90.7
**HApcs**					
Q_t_ (C)	284	515	760	955	1243
$n_{H_{2}}$ (μmol)	43,000	37,300	27,400	21,200	19,300
**CH-HApcs**					
Q_t_ (C)	83.3	117	121	99.3	78.9
$n_{H_{2}}$ (μmol)	416	690	743	499	345
X (%)	80.5	78.8	75.4	72.7	73.7
ρ_a_ (g/cm^3^)	0.088	0.110	0.113	0.111	0.144

**Table 3:** Number of exchanged electrons during the process of electrodeposition from chitosan-hydroxyapatite colloidal solution.

U(V)	8	12	16	20	24
z	2.15	1.76	1.70	2.06	2.37

## Data Availability

The data presented in this study are available on request from the corresponding author.

## References

[1] El Boraei N.F., Ibrahim M.A.M. (2019). Black binary nickel cobalt oxide nano-powder prepared by cathodic electrodeposition; characterization and its efficient application on removing the Remazol Red textile dye from aqueous solution. Mater. Chem. Phys..

[2] Waltman R.J., Diaz A.F., Bargon J. (1984). Substituent effects in the electropolymerization of aromatic heterocyclic compounds. J. Phys. Chem..

[3] Wang J., Wang L., Di J., Tu Y. (2009). Electrodeposition of gold nanoparticles on indium/tin oxide electrode for fabrication of a disposable hydrogen peroxide biosensor. Talanta.

[4] Cross E.R. (2020). The electrochemical fabrication of hydrogels: A short review. SN Appl. Sci..

[5] Besra L., Liu M. (2007). A review on fundamentals and applications of electrophoretic deposition (EPD). Prog. Mater. Sci..

[6] Geng Z., Wang X., Guo X., Zhang Z., Chen Y., Wang Y. (2016). Electrodeposition of chitosan based on coordination with metal ions: In situ -generated by electrochemical oxidation. J. Mater. Chem. B.

[7] Nawrotek K., Tylman M., Rudnicka K., Balcerzak J., Kamiński K. (2016). Chitosan-based hydrogel implants enriched with calcium ions intended for peripheral nervous tissue regeneration. Carbohydr. Polym..

[8] Nawrotek K., Tylman M., Decherchi P., Marqueste T., Rudnicka K., Gatkowska J., Wieczorek M. (2016). Assessment of degradation and biocompatibility of electrodeposited chitosan and chitosan–carbon nanotube tubular implants. J. Biomed. Mater. Res. Part A.

[9] Santosa K.B., Oliver J.D., Cederna P.S., Kung T.A. (2020). Regenerative Peripheral Nerve Interfaces for Prevention and Management of Neuromas. Clin. Plast. Surg..

[10] Reyes O., Sosa I., Kuffler D.P. (2005). Promoting neurological recovery following a traumatic peripheral nerve injury. P. R. Health Sci. J..

[11] Nawrotek K., Tylman M., Adamus-Włodarczyk A., Rudnicka K., Gatkowska J., Wieczorek M., Wach R. (2020). Influence of chitosan average molecular weight on degradation and stability of electrodeposited conduits. Carbohydr. Polym..

[12] Nawrotek K., Tylman M., Rudnicka K., Gatkowska J., Wieczorek M. (2016). Epineurium-mimicking chitosan conduits for peripheral nervous tissue engineering. Carbohydr. Polym..

[13] Kim E., Xiong Y., Cheng Y., Wu H.C., Liu Y., Morrow B.H., Ben-Yoav H., Ghodssi R., Rubloff G.W., Shen J. (2015). Chitosan to connect biology to electronics: Fabricating the bio-device interface and communicating across this interface. Polymers.

[14] Pang X., Zhitomirsky I. (2005). Electrodeposition of composite hydroxyapatite-chitosan films. Mater. Chem. Phys..

[15] Haastert-Talini K., Geuna S., Dahlin L.B., Meyer C., Stenberg L., Freier T., Heimann C., Barwig C., Pinto L.F.V., Raimondo S. (2013). Chitosan tubes of varying degrees of acetylation for bridging peripheral nerve defects. Biomaterials.

[16] Koev S.T., Dykstra P.H., Luo X., Rubloff G.W., Bentley W.E., Payne G.F., Ghodssi R. (2010). Chitosan: An integrative biomaterial for lab-on-a-chip devices. Lab Chip.

[17] Cheng Y., Luo X., Betz J., Buckhout-White S., Bekdash O., Payne G.F., Bentley W.E., Rubloff G.W. (2010). In situ quantitative visualization and characterization of chitosan electrodeposition with paired sidewall electrodes. Soft Matter.

[18] Cheng Y., Gray K.M., David L., Royaud I., Payne G.F., Rubloff G.W. (2012). Characterization of the cathodic electrodeposition of semicrystalline chitosan hydrogel. Mater. Lett..

[19] Gebhardt F., Seuss S., Turhan M.C., Hornberger H., Virtanen S., Boccaccini A.R. (2012). Characterization of electrophoretic chitosan coatings on stainless steel. Mater. Lett..

[20] Nguyen T.T.B., Hein S., Ng C.H., Stevens W.F. (2008). Molecular stability of chitosan in acid solutions stored at various conditions. J. Appl. Polym. Sci..

[21] Il’ina A.V., Varlamov V.P. (2004). Hydrolysis of chitosan in lactic acid. Appl. Biochem. Microbiol..

[22] Danilchenko S.N. (2009). Chitosan–hydroxyapatite composite biomaterials made by a one step co-precipitation method: Preparation, characterization and in vivo tests. J. Biol. Phys. Chem..

[23] Manoj M., Mangalaraj D., Ponpandian N., Viswanathan C. (2015). Core-shell hydroxyapatite/Mg nanostructures: Surfactant free facile synthesis, characterization and their in vitro cell viability studies against leukaemia cancer cells (K562). RSC Adv..

[24] Khachani M., El Hamidi A., Halim M., Arsalane S. (2014). Non-isothermal kinetic and thermodynamic studies of the dehydroxylation process of synthetic calcium hydroxide Ca(OH)2. J. Mater. Environ. Sci..

[25] Madhusudanan P., Raju G., Shankarappa S. (2020). Hydrogel systems and their role in neural tissue engineering. J. R. Soc. Interface.

[26] George P.M., Saigal R., Lawlor M.W., Moore M.J., LaVan D.A., Marini R.P., Selig M., Makhni M., Burdick J.A., Langer R. (2009). Three-dimensional conductive constructs for nerve regeneration. J. Biomed. Mater. Res. Part A.

[27] Abidian M.R., Daneshvar E.D., Egeland B.M., Kipke D.R., Cederna P.S., Urbanchek M.G. (2012). Hybrid Conducting Polymer-Hydrogel Conduits for Axonal Growth and Neural Tissue Engineering. Adv. Healthc. Mater..

[28] Venkatesan J., Kim S.K. (2010). Chitosan composites for bone tissue engineering—An overview. Mar. Drugs.

[29] Bonferoni M.C., Giunchedi P., Scalia S., Rossi S., Sandri G., Caramella C. (2006). Chitosan gels for the vaginal delivery of lactic acid: Relevance of formulation parameters to mucoadhesion and release mechanisms. AAPS Pharm. Sci. Tech..

[30] de Soares L.S., Perim R.B., de Alvarenga E.S., de Guimarães L.M., de Teixeira A.V.N.C., dos Coimbra J.S.R., de Oliveira E.B. (2019). Insights on physicochemical aspects of chitosan dispersion in aqueous solutions of acetic, glycolic, propionic or lactic acid. Int. J. Biol. Macromol..

[31] Yan K., Ding F., Bentley W.E., Deng H., Du Y., Payne G.F., Shi X.W. (2014). Coding for hydrogel organization through signal guided self-assembly. Soft Matter.

[32] Simchi A., Pishbin F., Boccaccini A.R. (2009). Electrophoretic deposition of chitosan. Mater. Lett..

[33] Ducheyne P., Kim C.S., Pollack S.R. (1992). The effect of phase differences on the time-dependent variation of the zeta potential of hydroxyapatite. J. Biomed. Mater. Res..

[34] Zhitomirsky I., Gal-Or L. (1997). Electrophoretic deposition of hydroxyapatite. J. Mater. Sci. Mater. Med..

[35] Katí J., Metikoš-Huković M., Škapin S.D., Petravíć M., Varašanec M. (2014). The potential-assisted deposition as valuable tool for producing functional apatite coatings on metallic materials. Electrochim. Acta.

[36] Li T.T., Ling L., Lin M.C., Jiang Q., Lin Q., Lou C.W., Lin J.H. (2019). Effects of ultrasonic treatment and current density on the properties of hydroxyapatite coating via electrodeposition and its in vitro biomineralization behavior. Mater. Sci. Eng. C.

[37] Izquierdo R., Garcia-Giralt N., Rodriguez M.T., Cáceres E., García S.J., Gómez Ribelles J.L., Monleón M., Monllau J.C., Suay J. (2008). Biodegradable PCL scaffolds with an interconnected spherical pore network for tissue engineering. J. Biomed. Mater. Res. Part A.

